# One-stop clinic for patients with suspected ovarian cancer: results from a retrospective outcome study of the referral pathway

**DOI:** 10.1186/s12905-021-01540-w

**Published:** 2021-12-28

**Authors:** Ayisha A. Ashmore, Chellappah Gnanachandran, Iqra Luqman, Kathryn Horrocks

**Affiliations:** Department of Gynaecology, Northampton General Hospital, Cliftonville, Northampton, NN1 5BD UK

**Keywords:** Ultrasound scanning, Ovarian cancer, Cancer screening, Tumour markers, Clinical effectiveness

## Abstract

**Background:**

Women with abdominal pain and bloating frequently have their Ca-125 levels investigated for suspected ovarian cancer and this has led to a significant increase in referrals to the ovarian cancer service. We have conducted this study to help improve the efficiency in which these patients are investigated and to improve future pathways within the referral service.

**Methods:**

This was a retrospective observational outcome study. Data were collected from electronic documents of patients’ referrals, assessments, and clinical correspondences over 48 months. The study was conducted in a secondary gynaecology cancer centre with direct referrals from primary care. The pelvic mass clinic was set up to include a consultation and an ultrasound scan with support available for patients if required. All patients included were referred directly from primary care for suspected ovarian cancer with Ca-125 result over a period of 2 years.

**Results:**

286 were referred from primary care according to the NICE guidelines of ‘2-week wait for ovarian cancer’. Only 223 patients who had a Ca-125 result reported at the time of their referral were included in the analysis. Out of the 223 patients, 126 patients were discharged with or without a repeat Ca-125 after the initial assessment. 18 patients were diagnosed with cancer following the referral, but only 12 of them had a primary ovarian malignancy. The malignancy rate in women under 50 years of age was 22% (4/18) and 78% (14/18) in women aged 50 or above.

**Conclusion:**

One-stop focused gynaecology ultrasound clinics where clinicians may assess patients and perform ultrasound scans for suspected cancer, may be better for managing this patient population due to improved efficiencies in waiting times, same day diagnosis and a reduction in waiting times to first appointment. Secondly, the majority of the patients with Ca-125 of more than 35 U/mL, who were referred through this pathway, did not have cancer. This review queries the future value of using Ca-125 as the basis for referrals from primary care for suspected ovarian malignancy. Further studies are required to assess whether a higher Ca-125 cut off may be used as the basis of referrals for premenopausal women.

## Background

Around 7000 women are diagnosed with ovarian cancer every year in the UK with a significant proportion presenting with advanced disease [1]. The average, 5-year survival rate in the UK is 46%, which is lower than the European 5-year survival rate for ovarian cancer. The early diagnosis of primary ovarian malignancy is paramount in increasing the rate of 5-year survival. For example, when diagnosed in the initial stages, 90% of women will survive for at least 5 years, contrasted to a 5-year survival of under 50% when diagnosed in the latest stage [1].

The NICE referral pathway for primary care for suspected ovarian cancer has recommended a Ca-125 measurement as part of the ‘2-week wait suspected cancer pathway’ in 2011, 2015 and 2017 [2, 3]. NICE adopted Ca-125, which has long been associated with female reproductive tract pathology, as a tool to identify women at risk.

Ca-125 is a glycoprotein which is a component of the gastrointestinal tract, the respiratory tract, and the female reproductive tract. Since its discovery in 1981, serum Ca-125 has been associated with both benign and malignant female reproductive tract pathology [4, 5]. Raised Ca-125 is associated with ovarian cancer and has been used as a serum marker for ovarian cancer [6]. Preoperative Ca-125 is an independent risk factor and helps in decision-making models [7–9]. However, marginally increased levels are not disease-specific and are poorly correlated with the diagnosis of ovarian malignancy [4, 6, 10]. Besides, serum Ca-125 is frequently within normal limits seen in some early ovarian cancers.

Nationally there has been a drive to promptly refer patients for further investigation and treatment for suspected ovarian cancer. The introduction of Ca-125 as a referral criterion has significantly increased the number of referrals for further investigation. Current referral criteria have resulted in an enormous increase in demand for the investigation of suspected gynaecological malignancies leading to anxiety and stress for patients.

Although one-stop clinics have been used in the diagnosis of cancers in other specialties as well as routinely within gynaecology [11–18], our study is possibly the first to review the effectiveness and efficiency of a one-stop focused gynaecology ultrasound clinic (OSFGUC) for suspected ovarian cancers. In our clinic, when a patient is referred via the primary care pathway, they are reviewed initially in the OSFGUC for complete assessment by a consultant gynaecologist. Our practice involves using ultrasonography to fully assess patients as part of the initial examination, thereby expediting diagnosis and enhancing the patient pathway.

## Methods

We conducted a retrospective outcome-based observational study using data from electronic documents at Northampton General Hospital. The OSFGUC involves a consultation, including sonographic assessment by a consultant gynaecologist, followed by the formulation of a plan for discharge, further investigation, or surgery.

Data were collected from all patients referred from the region to the 2-week wait clinic for suspected ovarian cancer over 2 years (August 2016–August 2018). Patients, who were referred from other regional hospitals for treatment of adnexal masses or confirmed diseases were excluded. The total number of patients seen through direct ovarian cancer 2-week wait pathway was 286. All included patients were referred via the 2-week wait pathway.

Patients included in the study had symptoms such as pain, bloating or discomfort with or without a raised Ca-125. Patients who were referred with only a raised Ca-125 without prominent ovarian cancer symptoms (asymptomatic or symptoms not clearly defined but their GP had performed a Ca-125 test) were also included in the study analysis (n = 125). Patients with a palpable pelvic mass but no Ca-125 result at the time of referral were excluded. The total number of patients included in our analysis was 223.

Our outcome measures included discharge after the first appointment, scheduled follow-ups, operative intervention, and final histologic diagnosis following surgery.

Although data based on the IOTA principles [19] were collected, an analysis was not performed due to low numbers of patients for conclusions.

## Results

The total number of patients included in our analysis was 223. One hundred and forty-four (144) patients were aged 50 years or above (65%). Seventy-nine (79) patients were aged below 50 (35%).

The primary reason for referral to the pathway (Table 1: Reason for referral based on age group) was the identification of an elevated serum Ca-125, by a primary care physician, along with either pelvic pain, discomfort or sensation of abdominal/pelvic mass.

**Table 1:** Reason for referral based on age group

Reason for referral	Age < 50	Age 50 and above	Grand total
Bloating	0	9	9
Raised Ca-125 (> 35 U/mL) without specific symptoms defined	44	81	125
Incidental cyst	2	11	13
Mass	17	28	45
Pain	9	10	19
Other	7	5	12
Grand Total	79	144	223

An elevated serum Ca-125 was observed in 81 out of 144 patients (56%) aged 50 or over and in 44 out of 79 patients (56%) aged below 50. Therefore a total of 125 patients were asymptomatic. The remaining patients, in both age groups, were referred to the clinic with specific symptoms only and were found to have a Ca-125 below 35U/mL (Table 2: Ca-125 level based on age group).

**Table 2:** Ca-125 level by age group

Ca-125 level (U/mL)	Age < 50	Age 50 and above	Grand total
Normal (< 35)	31	57	88
35–69	48	87	135
70–99	26	38	64
> 100	13	28	41
Grand total	79	144	223

Forty-four patients (20%) had surgical interventions following their initial consultation and ultrasound scan in one-stop clinic (OSC), with or without further imaging. Fifteen patients underwent laparoscopic surgeries (including salpingo-oophorectomy or ovarian cystectomy). Twenty-five patients underwent major surgeries (such as total abdominal hysterectomy ± bilateral salpingo-oophorectomy and omentectomy). Only four patients had a total laparoscopic hysterectomy or open hysterectomy for benign disease. One hundred and twenty six patients (56%) were discharged directly from clinic with or without a repeat Ca-125 test.

Of 223 patients included in the study, only 18 (8%) were diagnosed with a malignancy which was confirmed histologically. Out of these 18, only 12 patients were identified as having a primary ovarian malignancy. The other six malignancies were primary cancers from the gastrointestinal tract or metastases from other abdominal primary cancers (Table 3: histological outcome based on Ca-125).

**Table 3:** Histological outcome based on Ca-125

Histological outcome	Ca-125 < 35 U/mL		Ca-125 > 35 U/mL		Grand total
	Age < 50	Age 50 and above	Age < 50	Age 50 and above	
Benign pelvic disorders (including inflammatory disorders)	5	10	9	5	29
Benign uterine disorders (including fibroids)	9	6	9	14	38
Uterine malignancy	0	0	0	1	1
Ovarian malignancy	2	3	1	6	12*
Abdominal malignancy but not related to reproductive organs	0	2	1	2	5
No significant pelvic or abdominal pathology	15	36	28	59	138
Grand total	31	57	48	87	223

^*^2 Borderline, 1 Germ-cell tumour, 1 Struma Ovarii, 1 FIGO 1a

We performed further analysis (see Tables 3, 4 and 5) to consider whether a higher cut-off value for referrals using Ca-125 would lead to fewer malignancies being identified. Increasing the referral threshold value of Ca-125 from 35 U/mL to either 70 or 100 U/mL was retrospectively explored to identify the rate of missed malignancies.

**Table 4:** Histological outcome based on Ca-125 > 70 U/mL

Histological outcome	Ca-125 < 70 U/mL	Ca-125 > 70 U/mL	Grand total
Benign pelvic disorders (including inflammatory disorders)	17	13	30
Benign uterine disorders (including fibroids)	25	13	38
Uterine malignancy	0	1	1
Ovarian malignancy	5* (see above of the types and stage)	7	12
Abdominal malignancy but not related to reproductive organs	2	3*	5
No significant pelvic or abdominal pathology	100	37	137
Grand total	150	74	223

**Table 5:** Histological outcome Ca-125 > 100 U/mL

Histological outcome	Ca-125 < 100 U/mL	Ca-125 > 100 U/mL	Grand total
Benign pelvic disorders (including inflammatory disorders)	26	4	30
Benign uterine disorders (including fibroids)	33	5	38
Uterine malignancy	1		1
Ovarian malignancy	10	2	12
Abdominal malignancy but not related to reproductive organs	4	1	5
No significant pelvic or abdominal pathology	101	36	137
Grand total	176	48	223

We found that at the level of Ca-125 of 70 U/mL, one non-ovarian cancer would not have been referred, however, if a value of 100 U/mL were used, five additional malignancies would have been missed.

## Discussion

Our study is the first to report on the outcomes of an ovarian cancer referral pathway from the community to secondary care based on one-stop focused gynaecology ultrasound clinic (OSFGUC). The OSFGUC clinic not only expedites the diagnosis of malignancy but may alleviate patient anxiety when being investigated for suspected malignancy if ultrasound scans are not suggestive of sinister pathology. Studies have been conducted where an assessment of patient satisfaction and anxiety levels have taken place. There are varied reports which suggest that although patient satisfaction may improve due to same-day results, perceived patient centred care and continuity of carer [20], one stop clinics may not be justified for the purpose of reducing short term anxiety [21, 22]. However, we would argue that OSCs allow for clinician delivered ultrasound scans and so bring the added value of clinical interpretation of high quality ultrasound which could allay ongoing patient anxieties in suspected ovarian cancer. Although these outcomes were not prospectively collected in our study, they would greatly strengthen the case for OSFGUCs and could help direct policy makers to the benefits to patient experience that OSCs hold.

We found that our one-stop focused gynaecology ultrasound clinic (OSFGUC) was highly effective in the management of an increased patient burden by way of offering a service that provides same-day full assessment, definitive diagnosis, and management plan. As the results suggest, almost two-thirds of patients were able to be discharged directly from the clinic following their initial appointment followed up by primary care for repeat Ca-125 test. These results are echoed by findings from studies into other cancer services. Friedemann Smith et al. have performed a systematic review on the use of of OSCs. They found that OSC’s were associated with a significant reduction in waiting times between referral and clinic appointment and a significantly increased proportion of patients with same day diagnoses [23]. However, the authors commented that the appropriateness of referrals to one-stop clinics relative to usual care clinics were not assessed. This element may contribute to inappropriate referrals and burden on one-stop clinics and so strict criteria may have to be developed in order to maintain the efficiencies gained from this model of service provision. That being said, Sorelli et al. reported that the majority of referrals from primary care to flexible sigmoidoscopy clinic were appropriate [12]. In gynaecology itself, multiple studies have been conducted to assess the throughput of OSCs [15–18]. All have reported improvements in hospital waiting lists and throughput of patients in postmenopausal bleeding pathways. As such, we feel that some of these efficiencies could be translatable to ovarian cancer pathways.

Our study has demonstrated that raising the referral criteria from a Ca-125 of 35–70 U/mL, for the suspected ovarian cancer pathway, reduced the number of patients who may not have needed a direct referral to cancer services. We feel that these patients should be referred, instead, for a pelvic ultrasound to exclude pelvic pathology. If a new cut-off of 70 U/mL, particularly for premenopausal women, was implemented, a significant proportion of patients would not be referred to the suspected cancer clinic. By using a higher value of Ca-125 for premenopausal women as a threshold for referral, we may reduce the burden on gynaecology services. A higher threshold of Ca-125 for referral, in turn, prevent unnecessary, anxiety-inducing referrals of young women to gynaecology clinics with conditions such as endometriosis. A recent study by Fulston et al. supports the findings from our study [24]. The authors report a Ca-125 value of 104 U/mL in a 40 year old woman was the equivalent of a value of 32 U/mL in a 70 year old woman in their dataset. This would support our recommendation that a higher cut-off value may be used in premenopausal women.

While our approach could be replicated in other centres and units for gynaecology malignancies, several limitations must be considered.

Our study has a small sample size and therefore lacks generalisability. We are currently collecting further data to strengthen our conclusions with regards to this matter. Further, if our model could be replicated, a clinic with ultrasound scanning facilities would be required with training on scanning and accreditation for gynaecology scanning.

While our model for ovarian cancer pathway demonstrates a more streamlined and rapid assessment of the discussed cohort of patients, it does not necessarily follow that such a service offers value for money. One study was identified where an economic evaluation was performed comparing OSC to traditional clinics in breast care [21]. The study found that the OSC model was more expensive than a traditional clinic but attributed this cost to the additional input of radiologists and pathologists. However this cost was offset by the reduction in number of prediagnostic visits. A cost analysis would be useful in determining how efficient this pathway would be in comparison to current practice for suspected ovarian cancer.

Finally, for patients who had high Ca-125 but normal pelvis on the scan, a repeat Ca-125 test was performed if needed by their general practitioner. If Ca-125 continued to rise, then they were re-referred to clinic for further advice and management. We aim to report the number of patients who needed further follow up and their 2 years outcome in our subsequent studies.

The lack of specificity of Ca-125, especially in premenopausal women and validity of ultrasonic features of adnexal mass for prediction of malignancy is well-documented [4, 19, 25]. We recommend that patients with symptoms suspicious of ovarian cancer should be referred to a clinic where ultrasound scan facilities are available as part of the initial consultation. Further research is also required to establish whether a higher referral threshold value for Ca-125 is safe if ultrasound facilities are available for normal to marginally high Ca-125.

## Conclusions

The one-stop focused gynaecology ultrasound clinic (OSFGUC) has been instrumental in helping to manage a substantial number of referrals and  can also provide a timely diagnosis of ovarian pathology. It permits a vast majority of patients to be discharged from the 2-week wait pathway back to the primary care with further monitoring of tumour markers and or repeat scan in some patients. The benefits of an OSC approach to ovarian cancer may include improving patient satisfaction, reducing anxiety related to awaiting results, reduce the time taken to diagnosis and improve the throughput of patients in the pathway. This is of particular importance given the need to reduce patient exposure to healthcare settings during the Covid-19 pandemic and OSC’s will reduce the number of times patients need to attend healthcare setings for assessment. For ovarian cancers specifically, ultrasound assisted OSCs have the additional benefit of adding experienced clinical interpretation of high quality ultrasound leading to sensible and safe decision making and initiation of management if required.

Secondly, at present, NICE guidance suggests that a serum Ca-125 > 35U/mL should be the threshold at which a patient is referred from primary care to gynaecology services. The optimal threshold for referrals has never been determined [4]. Future studies should explicitly examine the correlation of serum Ca-125, in conjunction with symptomology and ultrasonography, with final histologic diagnoses. This would better characterise the role of this tumour marker in identifying those at high risk of ovarian malignancy and would permit further optimisation of the primary care referral process.

The UK ROckETS study [26] may be able to answer several questions regarding the combined use Ca-125, other biomarkers and IOTA principles-based ultrasound scans in characterising the individual risk of ovarian malignancy. Developing a validated risk scoring model will optimise the referral process, reduce clinic burden and fast track the patients who need urgent treatment.

## Data Availability

The datasets used and/or analysed during the current study are available from the corresponding author on reasonable request.

## Abbreviations

NICE: National institute for health and care excellence
OSFGUC: One-stop focused gynaecology ultrasound clinic
OSC: One-stop clinic
